# Diabetes-related mortality in the immigrant compared to the Italian population, before and during the pandemic

**DOI:** 10.1007/s40618-025-02582-9

**Published:** 2025-08-26

**Authors:** Ugo Fedeli, Enrico Grande, Francesco Grippo, Gianni Corsetti, Giacomo Zoppini

**Affiliations:** 1 Epidemiological Department, Azienda Zero, Veneto Region, Padova, Italy; 2 Integrated System for Health, Social Assistance and Welfare, Italian National Institute of Statistics, Rome, Italy; 3 Population Register, Demographic and Living Conditions Statistics, Italian National Institute of Statistics, Rome, Italy; 4 Division of Endocrinology, Diabetes and Metabolic Diseases, Department of Medicine, University and Hospital Trust of Verona, Verona, Italy; 5 Endocrinology, Diabetes and Metabolism, Department of Medicine, University and University Hospital of Verona, Piazzale Stefani, 1, Verona, 37126 Italy

**Keywords:** Diabetes mortality, Immigrants, Multiple causes of death

## Abstract

**Purpose:**

A sharp increase in diabetes-related mortality has been registered during the pandemic. Although immigrants are known to suffer from a higher prevalence of diabetes than host populations, data on diabetes-related mortality in pandemic years among immigrants are lacking.

**Methods:**

All deaths with any mention of diabetes (multiple causes of death-MCOD) among subjects aged 20–64 years were extracted from the Italian National Cause of Death Register in the years 2019–2021. Directly age-standardized mortality rates (2013 European standard population) were computed. Standardized mortality ratios (SMR) for different immigrant groups were estimated with expected numbers based on rates registered among Italian citizens.

**Results:**

Overall age-standardized mortality rates related to diabetes increased by 30% in 2020 compared to 2019, remaining high in 2021. Before the pandemic, large differences were observed across different immigrant groups; during the pandemic, the mortality disadvantage versus the native population widened among those already at increased risk. Among female immigrants, SMR in 2021 were 1.9 (95% Confidence Interval 1.1–2.8) in North Africans, 4.4 (2.5–6.3) in Sub-Saharan Africans and 2.8 (1.6–4.1) in South Asians.

**Conclusion:**

Surveillance based on MCOD is warranted to assess if the large differences in diabetes-related mortality observed across different populations living in Italy will reduce in the next years.

**Supplementary Information:**

The online version contains supplementary material available at 10.1007/s40618-025-02582-9.

## Introduction

During the pandemic’s first year, a sharp increase in mortality associated to diabetes reversed the pre-existing declining long-term trend in Italy [1]. Such increase was more comprehensively measured by analyses extended to any mention of the disease in death certificates (multiple causes of death– MCOD approach), rather than by standard statistic, limited to the underlying cause of death (UCOD) [2]. During the second year of the pandemic, the excess diabetes-related mortality was reduced among the elders, who were involved in the early phases of the vaccination campaign, but persisted among the population aged 40–79 years [3]. The excess diabetes-related mortality based on MCOD was larger than the increase in all-cause mortality, a finding similar to reports on national data in Spain [4] and the US [5]. In the US, the increase in diabetes-related mortality was larger in the population aged 20–64 years [6], and among Hispanics, Blacks, and Asians compared to non-Hispanic Whites [6, 7].

Several factors suggest that immigrants in Italy might have represented a population group at a disproportionally high risk of diabetes-related mortality during the pandemic. In fact, the prevalence of known diabetes is higher in most immigrant groups than in the Italian population, with the highest rate found in immigrants from South Asia, followed by those form Northern and Sub-Saharan Africa [8–10]. This finding should also be interpreted in view of the evidence reported mainly from the US of a higher proportion of undiagnosed diabetes in the immigrant with respect to the native population [11]. Furthermore, immigrants constituted a vulnerable population during the pandemic in Italy, with higher excess mortality compared to the native population, especially among immigrants born in non-European countries with strong migratory pressure [12]. Higher COVID- 19 overall hospitalization and intensive care unit admissions were also observed with respect to Italians [13]. Moreover, the effect of diabetes on the risk of infection and death from COVID- 19 was slightly higher in immigrants [14]. In the later phases of the pandemic, low vaccination rates were an additional disadvantage factor: at the end of 2021, first-dose vaccination coverage was higher among Italian citizens aged 40–59 years (about 90%) compared with immigrants groups, with values ranging from 60 to 80% [15].

In spite of all the above, recent data on diabetes-related mortality rates according to immigrant status are completely lacking. In previous studies from European countries, mortality from diabetes was generally higher in migrant compared to local-born populations [16]; however, data were limited to the UCOD and dated back to the 1990–2000 s. Aim of the report is to investigate variations in diabetes-related mortality across different immigrant groups at the national level in Italy before the pandemic, and to assess changes registered in 2020–2021.

## Methods

Analyses were carried out on the Italian National Cause of Death Register, managed by the Italian National Institute of Statistics (ISTAT), which collects death certificates for all deaths occurring in Italy. All conditions reported on the death certificate were coded according to the International Classification of Diseases, 10 th Revision (ICD- 10). Analyses based on the MCOD approach were carried out to fully assess the burden of mortality associated with diabetes: to this purpose, all deaths occurring from January 2019 to December 2021 among residents in Italy with any mention of diabetes (ICD- 10 codes E10-E14) were extracted. Age‐standardized rates both for overall mortality and for mortality related to diabetes (direct method, European standard population- 2013 revision) were computed for all residents, and broken down by country of citizenship grouped by macro-geographical regions, as defined in previous studies on diabetes prevalence and causes of mortality in the immigrant population [8, 17]. Since the immigrant population is scarcely represented among older age groups, all analyses were restricted to subjects aged 20–64 years. In fact, the share of subjects aged ≥ 65 years in 2020 was about 25% among residents with Italian citizenship, but 5% or lower across immigrant groups, except for those from high-income countries (Table 1). Furthermore, an additional standardization approach was also adopted: standardized mortality ratios (SMR) were computed in each study year as the ratios between deaths observed across immigrant groups and those expected according to age- and sex-specific mortality rates registered among Italian citizens [17]. Such indirect standardization allows only for comparisons between each immigrant group and the Italian population taken as the reference, but is more robust in the presence of small numbers and of large unbalances in population age structures. 95% confidence intervals (CI) based on the Poisson distribution were calculated by the Byar’s approximation. All the analyses were carried out on mortality data routinely collected by ISTAT, therefore the study was exempt from Institutional Review Board approval.

**Table 1:** Population by citizenship and age class, Italy, year 2020

Citizenship	Age class (years)					
	Total	< 20	20–34	35–49	50–64	> 64
	n	%	%	%	%	%
Italy	54,333,088	17.3	14.7	19.8	23.0	25.1
North Africa	674,507	29.7	21.5	31.4	12.8	4.7
Sub-Saharan Africa	454,410	19.8	41.6	27.2	9.7	1.8
South Asia	559,754	23.8	31.3	33.3	9.6	1.9
Other Asia	542,045	24.0	22.2	31.3	18.6	3.9
Central South America	358,898	18.2	25.5	33.5	17.6	5.1
Eastern Europe	2,298,133	21.1	23.0	31.0	19.7	5.3
High income	217,530	8.0	18.1	27.7	29.0	17.2
All areas*	59,438,828	17.7	15.6	20.8	22.5	23.4

*Includes 464 stateless or unspecified citizenship residents

## Results

**Fig. 1:**
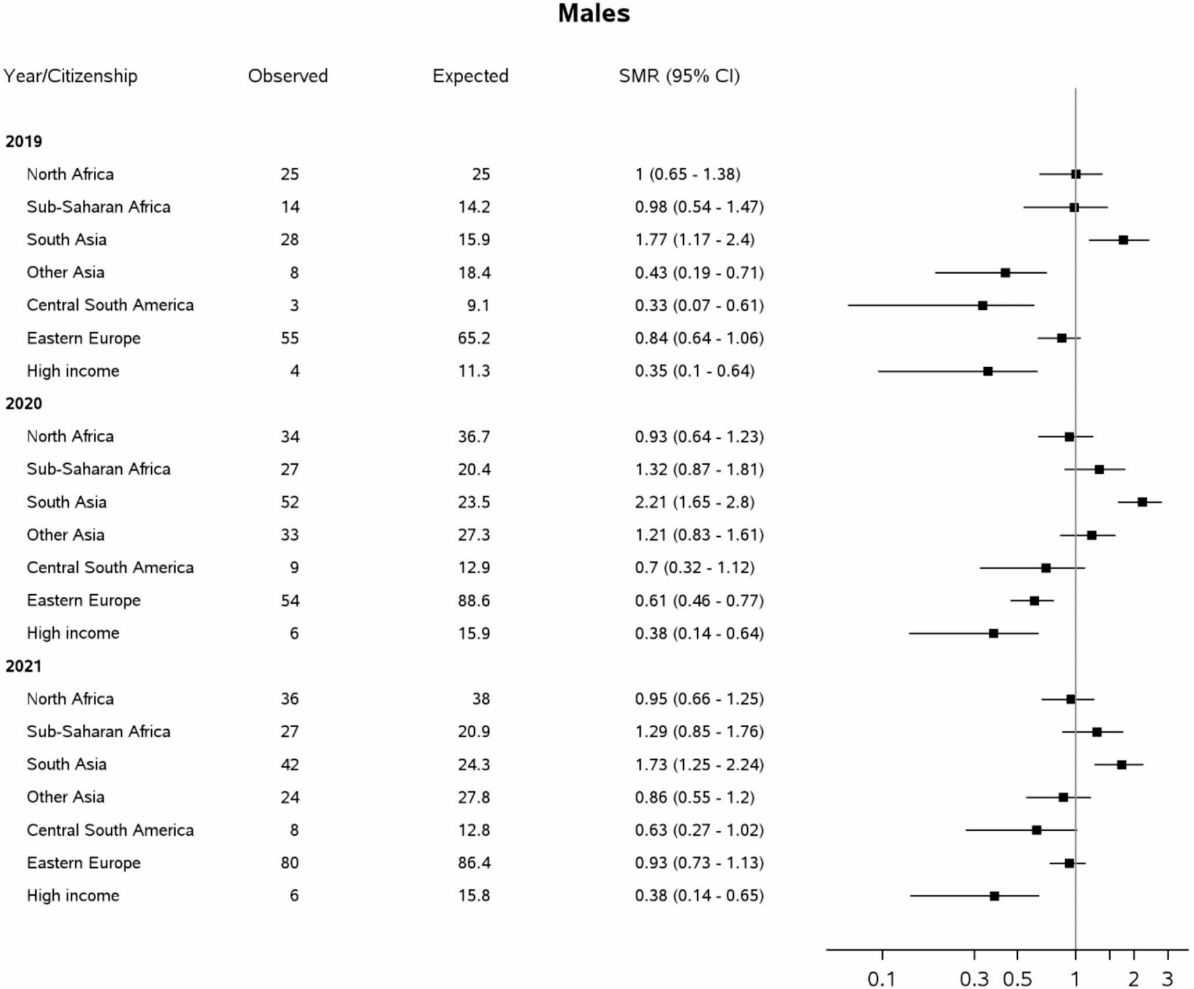
Standardized Mortality Ratios (SMR) for diabetes among foreign compared to Italian citizens aged 20–64 years. Italy, years 2019–2021, males

**Fig. 2:**
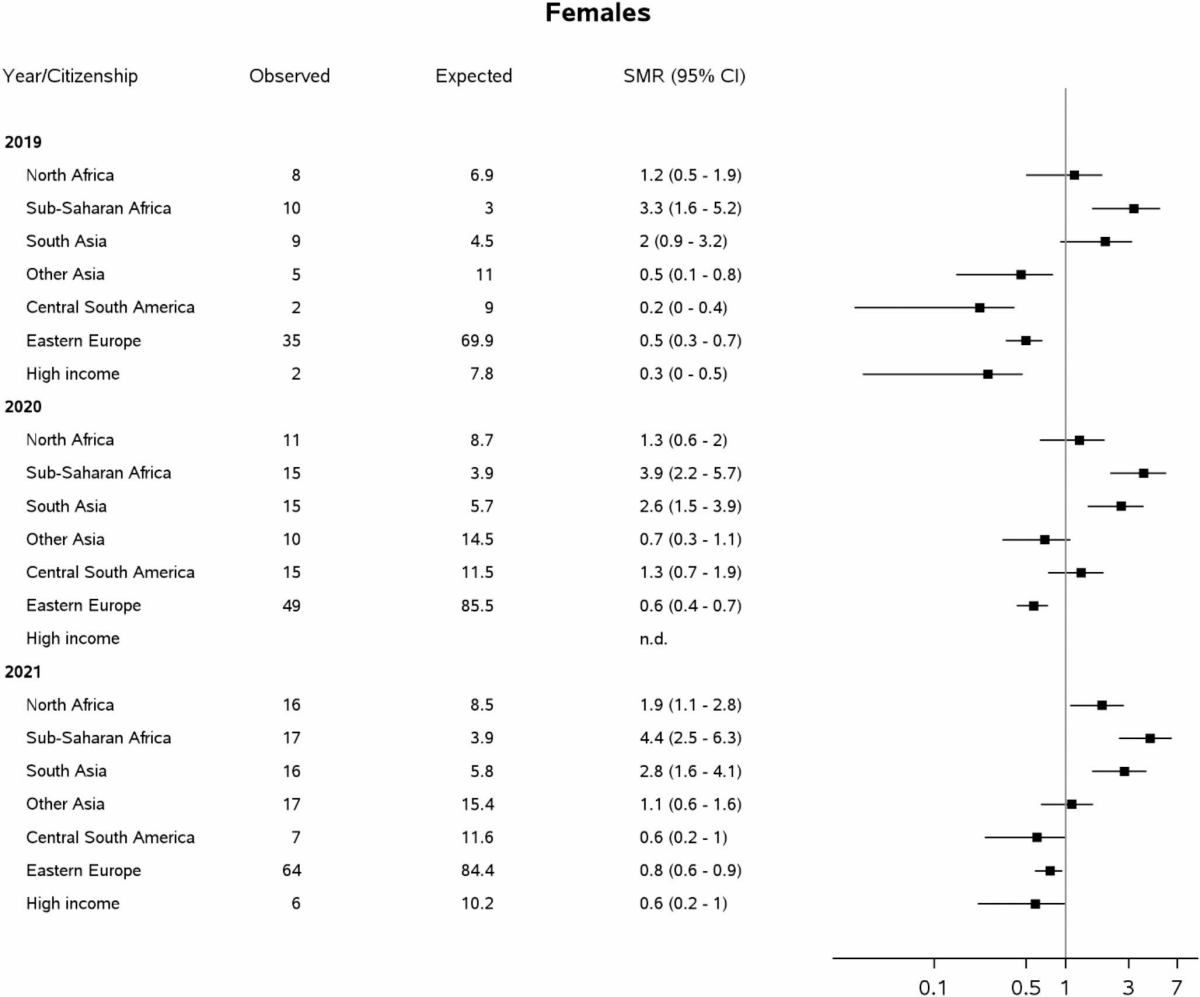
Standardized Mortality Ratios (SMR) for diabetes among foreign compared to Italian citizens aged 20–64 years. Italy, years 2019–2021, females. (High income 2020: only 1 observed death, SMR is not displayed)

In the overall population aged 20–64 years in Italy, 4,740 deaths with any mention of diabetes were registered in 2019, growing to 6,222 in 2020 and 6,162 in 2021. Age-standardized diabetes-related mortality increased by 30% in 2020 with respect to 2019 (+ 33% in males and + 22% in females), remaining almost unchanged in 2021. A peak in mortality was observed in 2020 among residents with South Asian citizenship, already affected by very high baseline rates (Table 2). The growth in diabetes-related mortality during the pandemic was much larger than that registered for all-causes mortality (overall + 10% in 2020, + 12% in males and + 6% in females, Supplementary Table 1). Taking the population with Italian citizenship as the reference, large differences were observed in the pre-pandemic year according to area of provenience: diabetes-related mortality was significantly higher among South Asians males and South Asians and Sub-Saharan African females (Figs. 1 and 2), similar among immigrants from North Africa, and lower in other immigrant groups. During the pandemic years, the mortality profile worsened among immigrants compared to Italians: the mortality advantage previously observed in some immigrants groups disappeared, whereas rates were significantly higher than those registered in Italians especially in females from North Africa, South Asia and Sub-Saharan Africa, among whom a twofold to fourfold increase in mortality risk was registered.

**Table 2:** Age-standardized mortality rates (per 100,000 residents, age 20–64 years) and number of deaths from diabetes in Italy, by sex and citizenship. Years 2019–2021

Citizenship	Males						Females						Males and Females					
	Age-adjusted rates (Number of deaths)																	
	2019		2020		2021		2019		2020		2021		2019		2020		2021	
Italy	17.4	(3141)	23.0	(4195)	22.2	(4091)	7.5	(1386)	9.0	(1681)	9.0	(1699)	12.4	(4527)	15.9	(5876)	15.5	(5790)
North Africa	19.7	(25)	23.1	(34)	20.3	(36)	10.4	(8)	10.7	(11)	19.2	(16)	15.9	(33)	18.1	(45)	20.0	(52)
Sub-Saharan Africa	12.2	(14)	27.1	(27)	26.1	(27)	24.7	(10)	35.3	(15)	41.2	(17)	15.7	(24)	29.6	(42)	30.1	(44)
South Asia	27.0	(28)	50.8	(52)	29.6	(42)	19.0	(9)	25.4	(15)	27.9	(16)	24.2	(37)	40.9	(67)	29.6	(58)
Other Asia	7.4	(8)	25.2	(33)	20.1	(24)	3.2	(5)	6.7	(10)	10.2	(17)	4.9	(13)	14.6	(43)	14.3	(41)
Central South America	6.1	(3)	12.1	(9)	10.3	(8)	1.8	(2)	12.4	(15)	5.9	(7)	3.0	(5)	12.6	(24)	7.4	(15)
Eastern Europe	14.7	(55)	14.4	(54)	20.6	(80)	3.7	(35)	5.3	(49)	6.7	(64)	6.7	(90)	7.8	(103)	10.5	(144)
High income	6.2	(4)	8.7	(6)	8.4	(6)	2.0	(2)	1.1	(1)	5.0	(6)	3.7	(6)	4.0	(7)	6.5	(12)
All areas*	17.3	(3282)	23.0	(4420)	22.2	(4317)	7.3	(1458)	8.9	(1802)	9.0	(1845)	12.2	(4740)	15.8	(6222)	15.5	(6162)

*Includes a total of 5 deaths of stateless or unspecified citizenship individuals

## Discussion

The study demonstrates a large increase in diabetes-related mortality among adults aged 20–64 during the pandemic’s first year in Italy, more evident among males and South Asians. The excess mortality persisted in 2021, with a more severe pattern in immigrant groups: the mortality gap versus the native population widened among those already at higher mortality risk, whereas mortality converged towards levels observed in the Italian population among those at lower risk before the pandemic.

The migrant may differ from the native population with regard to genetic, baseline risk distribution, lifestyle, health literacy and access to health care. All these may have played a consistent role in the worsening of mortality during the pandemic. Even though host societies promote behavioral changes among migrants, such as westernization of diet and a sedentary lifestyle, migrants may have a different baseline cardiovascular risks distribution, with large variations across different migrant populations. In fact, diabetes is more prevalent in some group such as South Asians, among whom diabetes tends to develop at an earlier age, a difference that may be due to a specific genetic susceptibility. The rates of cardiovascular diseases (CVD) and stroke, common causes of mortality among subjects with diabetes, were reported to be different among migrants groups: Middle East and South Asian migrants showed higher rates of CVD and stroke compared to the host populations, while migrants from Northern Africa had a lower rate of stroke [18]. In the Netherlands, South American migrants had higher stroke mortality, while North African migrants were at a lower mortality for CVD [19]. This latter finding was confirmed by a Spanish study, which reported lower rates in North African populations as compared to higher rates in Asian and sub-Saharan African groups [20]. Nevertheless, the host countries conditions are a further factor for differential risks: South Asian migrants were at an even higher CVD risk than their relatives in the country of origin [21]. In the RODAM (Research on Obesity & Diabetes among African Migrants) study, sub-Saharan African migrants in Europe showed higher rates of CVD than their counterparts living in a rural environment [22]. Furthermore, health literacy and access to health care are decisive issues in the successful treatment of chronic diseases such as diabetes and in cardiovascular prevention. Health literacy is fundamental to efficacious managing of chronic disease [23], but it is also central to prevention of infectious diseases. Prevention of complications of diabetes are a necessary step for reducing mortality [16], and treatment of diabetes is interdisciplinary and impacts heavily on the health care system [24]. In studies carried out in Italy before the pandemic, immigrants progressively represented a larger share of the population with diabetes [25], and displayed lower adherence to diagnostic and therapeutic protocols [10, 25].

Therefore, multiple mechanisms might explain the large impact of the pandemic on excess diabetes-related mortality in the immigrant population: the high prevalence of subjects with diabetes, at increased risk of severe COVID- 19; low vaccination rates, possibly influencing high mortality persisting in 2021; a disadvantaged socio-economic position, further exacerbated by job and income insecurity during the pandemic; limited health literacy and difficulties in the access to healthcare in an already over-whelmed healthcare system struggling to provide routine care for patients with chronic diseases [6].

The present nationwide study, although limited to only three years, included a large number of deaths among adults aged 20–64 years with diabetes (> 17,000) before and during the pandemic in a diverse population encompassing several ethnicities. Analyses extended to older age groups are warranted to fully assess the impact of the pandemic on diabetes-related mortality among the overall population. However, subjects aged ≥ 65 years are almost not represented in many immigrant groups (less than 2% of the population form Sub-Saharan Africa and South Asia). Furthermore, uncertainties in the denominator of mortality rates (e.g. due to unregistered re-emigration) increase among older age groups. In view of the above, also in previous studies comparing native and immigrants groups, both on diabetes prevalence and on causes of mortality, analyses were restricted to the adult population and excluded subjects aged above 60 or 65 years [8, 17].

To date, this is the first report investigating the impact of the pandemic on diabetes-related mortality across different immigrant groups. Although analyses restricted to the UCOD already demonstrated an increase in mortality from diabetes in many countries in 2020 [26], the availability of MCOD records represents a strength of the present study. In fact, when reported in the death certificate, diabetes is rarely selected as the UCOD (in about 25% of cases in Italy before the pandemic [27]); this issue has become of even greater importance in recent years, due to COVID- 19 acting as a strong competing condition for the selection as the UCOD. As a consequence, analyses extended to MCOD allowed for more fully estimating the impact of the pandemic on patients with diabetes, including both deaths attributed to COVID- 19 as the UCOD, and deaths attributed to diabetes, circulatory diseases and other causes [2, 6].

Study limits included the definition of immigrant groups based on citizenship, and the lack of data on length of residency in Italy and individual socioeconomic status [28]. Immigrants who acquired the Italian citizenship by marriage or after a long-term stay in Italy were classified in the present study as Italians in mortality rates both at the numerator (citizenship registered in death certificates) and at the denominator (resident population grouped by citizenship); this might slightly dilute the observed differences between population groups. Furthermore, the health status of immigrants is expected to converge towards that of the native population with increasing length of residency in the host country. Lastly, low socioeconomic status is associated with increased diabetes morbidity and mortality, and immigrants usually have a lower socioeconomic position; however, the large variation in mortality rates observed across ethnicity groups are unlikely to be accounted by socioeconomic differences [16].

In view of the present results, surveillance by means of the MCOD approach is warranted in the next years, to assess if the mortality profile related to diabetes will return to baseline levels across the different population groups living in Italy.

## Electronic supplementary material

Below is the link to the electronic supplementary material.

Supplementary Material 1

## Data Availability

Supporting data are available upon reasonable request to the ISTAT Contact Center.
